# *Coxiella burnetii* in domestic doe goats in the United States, 2019–2020

**DOI:** 10.3389/fvets.2024.1393296

**Published:** 2024-05-07

**Authors:** Halie K. Miller, Matthew Branan, Rachael A. Priestley, Raquel Álvarez-Alonso, Cara Cherry, Cody Smith, Natalie J. Urie, Alyson Wiedenheft, Clayton Bliss, Katherine Marshall, Gilbert J. Kersh

**Affiliations:** ^1^Rickettsial Zoonoses Branch, Centers for Disease Control and Prevention, Atlanta, GA, United States; ^2^United States Department of Agriculture, Animal and Plant Health Inspection Service, Center for Epidemiology and Animal Health, National Animal Health Monitoring System, Fort Collins, CO, United States; ^3^Animal Health Department, NEIKER-Instituto Vasco de Investigación y Desarrollo Agrario, Derio, Spain; ^4^Department of Clinical Sciences, Colorado State University, Fort Collins, CO, United States

**Keywords:** coxiellosis, Q fever, caprine, infectious disease, zoonosis

## Abstract

*Coxiella burnetii* is a bacterial pathogen capable of causing serious disease in humans and abortions in goats. Infected goats can shed *C*. *burnetii* through urine, feces, and parturient byproducts, which can lead to infections in humans when the bacteria are inhaled. Goats are important *C*. *burnetii* reservoirs as evidenced by goat-related outbreaks across the world. To better understand the current landscape of *C*. *burnetii* infection in the domestic goat population, 4,121 vaginal swabs from 388 operations across the United States were analyzed for the presence of *C*. *burnetii* by IS1111 PCR as part of the United States Department of Agriculture, Animal Plant Health Inspection Service, Veterinary Services’ National Animal Health Monitoring System Goats 2019 Study. In total, 1.5% (61/4121) of swabs representing 10.3% (40/388) (weighted estimate of 7.8, 95% CI 4.4–13.5) of operations were positive for *C*. *burnetii* DNA. The quantity of *C*. *burnetii* on positive swabs was low with an average Ct of 37.9. Factors associated with greater odds of testing positive included suspected Q fever in the herd in the previous 3 years, the presence of wild deer or elk on the operation, and the utilization of hormones for estrus synchronization. Factors associated with reduced odds of testing positive include the presence of kittens and treatment of herds with high tannin concentrate plants, diatomaceous earth, and tetrahydropyrimidines. *In vitro* analysis demonstrated an inhibitory effect of the tetrahydropyrimidine, pyrantel pamoate, on the growth of *C*. *burnetii* in axenic media as low as 1 μg per mL. The final multivariable logistic regression modeling identified the presence of wild predators on the operation or adjacent property (OR = 9.0, 95% CI 1.3–61.6, *p* value = 0.0248) as a risk factor for *C*. *burnetii* infection.

## Introduction

*Coxiella burnetii* is a zoonotic pathogen and the causative agent of Q fever. Reservoir hosts play an important role in *C*. *burnetii* transmission to humans and animals as copious amounts of the organism can be shed into the environment through contaminated feces, urine, milk, and parturition byproducts (1). Inhalation is the primary route of infection and ruminant livestock such as cattle, sheep, and goats are believed to be the major reservoirs, although a wide range of animals can become infected (1). Infected herds may go unnoticed due to minimal clinical signs, particularly for cattle; however, abortion storms are often the first sign of *C*. *burnetii* infection for sheep and goat herds (1). Infected placentas may contain as many as 10^9^ *C*. *burnetii* per gram of tissue, allowing for the introduction of high levels of infectious aerosols into the environment, not only following abortions, but also from live births as well (2–4). With an infectious dose for humans of less than 10 organisms, infected herds have the potential for widespread impact on public health (1). Environmental contamination with *C*. *burnetii* can be extensive and long lasting due to its ability to be carried long distances in the wind and withstand heat, desiccation, and UV exposure (1). In the United States there are no vaccines approved for human or animal use, nor are there approved therapies for the prevention and treatment of Q fever in domestic livestock. Furthermore, effective methods for eradication of *C*. *burnetii* from the environment are also lacking. Understanding how widespread *C*. *burnetii* is and how vulnerable the United States goat population is to *C*. *burnetii* infection is important for public health.

Infected goat herds were responsible for the largest Q fever outbreak in humans on record, which occurred in the Netherlands from 2007 to 2010 and resulted in over 4,000 acute infections (5). Acute Q fever is often marked by fever and symptoms frequently overlap with other respiratory pathogens such as influenza or severe acute respiratory syndrome coronavirus 2, which may lead to an underestimation of disease burden (1). Indeed, serological analysis following the Netherlands outbreak revealed an estimated 40,600 infections between 2007 and 2010 and impacted an estimated 15% of the population in the worst affected areas (5–7). Acute infections are typically self-limiting and a substantial number may be asymptomatic, although serious complications such as pneumonia and hepatitis can occur (1). *Coxiella burnetii* can persist in the human host for an extended period following the initial infection to erupt months to years later as a devastating chronic illness (1). Cases of chronic Q fever resulting from the 2007 to 2010 Netherlands outbreaks have been diagnosed at least 8 years after the initial outbreak (8). Chronic Q fever is a serious manifestation of *C*. *burnetii* infection that can present as fatal endocarditis (1). Other common manifestations of chronic Q fever include granulomatous hepatitis, osteomyelitis, and endovascular infection with atypical manifestations ranging from granulomatous lymphadenitis to abdominal aortic aneurysm (1).

Human cases of Q fever have resulted from domestic goat-associated outbreaks in the United States in Colorado, Washington, and Montana (9, 10). Despite the importance of goats as a reservoir for *C*. *burnetii* and the potential for widespread dissemination, the burden of disease in the United States domestic goat population is not well understood and testing is typically limited to outbreak events. To gain a better understanding of the current landscape of *C*. *burnetii* infection across goat herds in the United States, and to better inform public health measures, vaginal swabs were collected from goat does as part of the United States Department of Agriculture (USDA), Animal Plant Health Inspection Service, Veterinary Services’ National Animal Health Monitoring System (NAHMS) Goat 2019 Study (11). NAHMS conducts national studies on animal health, management, and productivity and the prior study in goats was carried out in 2009. NAHMS conducts studies on goats approximately every 10 years. Through this cross-sectional survey, 4,121 vaginal swabs were tested for the presence of *C*. *burnetii* DNA, an indicator of current infection. A risk factor analysis of herds positive for shedding of *C*. *burnetii* was carried out to assess operation characteristics and management practices associated with increased infection risk.

## Methods

### Study design and sampling strategy

#### Overview

This study was conducted as part of the USDA NAHMS Goat 2019 study, which occurred in the top 24 goat producing states. These states represented 75.8 percent of United States goat operations with five or more adult goats and 80.4 percent of goats on operations with five or more adult goats. Participating states were categorized into two regions: west [California, Colorado, Oregon, Washington, as well as portions of Oklahoma and Texas located west of interstate 35 (I-35)] and east (Alabama, Connecticut, Florida, Georgia, Indiana, Iowa, Kentucky, Michigan, Minnesota, Missouri, New York, North Carolina, Ohio, Pennsylvania, Tennessee, Vermont, Virginia, Wisconsin, as well as portions of Oklahoma and Texas located east of I-35). A map depicting the participating states and region descriptions can be found in the NAHMS Goat 2019 Descriptive Report I page 2 (11). Generally, NAHMS studies are designed with the prespecified precision criterion of achieving a coefficient of variation of 20% or less with 95% confidence for each of the sampling stratification variables for estimated proportions of 0.10 for the Phase I and Phase II questionnaire estimates. The number of operations sampled from the USDA National Agricultural Statistics Service (NASS) list frame was chosen to meet this criterion using a stratified random sample, with strata defined by region, herd size (5–19, 20–99, and 100 or more adult goats), and operation type (dairy, meat and other, and unknown), where large operations and dairy operations were selected at a higher rate than they appeared in the population to ensure that they were adequately represented in the sample. The sample sizes met or exceeded coefficient of variation design goal at each phase of the study. More information about the design of the NAHMS Goat 2019 study can be found in the NAHMS Goat 2019 Descriptive Report I (11).

#### Data collection

A total of 4,770 operations were sampled from the NASS list frame. NASS enumerators contacted sampled operations and asked producers to complete the Phase I questionnaire via personal interviews. Those who completed the Phase I questionnaire were asked if they would like to consent to the opportunity to be contacted by an APHIS-Veterinary Services (*VS*) veterinarian and/or animal health technician (AHT) to participate in Phase II of the study. Those who consented were contacted by APHIS-*VS* veterinarians and/or AHTs and were asked to complete the Phase II questionnaire via personal interviews. Those who completed the Phase II questionnaire had the opportunity to complete the vaginal swab biologics collection activities. The general goat management questionnaire and veterinary services questionnaires along with other material associated with the NAHMS Goat 2019 study can be accessed online (12). Samples from three operations that did not submit complete questionnaire data and 10 that were out of scope of the sample design were excluded from the analysis.

#### Sample procurement

Operations completing both the Phase I and Phase II questionnaires were eligible for biologics collection and testing. Because the NAHMS studies are voluntary, the number of operations participating in biologics collection is unknown at the design phase of the study. To make the most efficient use of biologics field data collection resources, the number of animals to sample from each operation was standardized across biologics specimen type and was estimated using projected response rates for previous phases of the study, estimated herd sizes of those operations, and estimated prevalence of the pathogens, diseases, and conditions of interest to meet the prespecified precision criteria. All does on participating operations were able to be sampled for the vaginal swab collection, up to 15 does, though three operations sampled up to 20 does.

Samples were collected from does on goat operations from September 2019 through January 2020, with most samples collected in October and November. Vaginal swabs were collected from does at least 15 months of age using sterile media-free rayon swabs (Becton, Dickinson). Veterinary medical officers and animal health technicians performed the sampling by inserting the swab gently at least halfway into the vagina and rotating 180 degrees, 4–5 times. Any swabs dropped on the ground were discarded. Pregnant does were included at the owner’s discretion.

#### Sample size

More vaginal swab samples were collected than were needed to achieve the prespecified precision criteria and the number of samples collected exceeded lab capacity. Therefore, a subset of vaginal swabs was chosen and sent to the lab for testing, where representativeness was enforced by herd size, region, primary production of the operation, state, and whether the operation experienced any abortions in the previous 12 months, ensuring that the subset sample size still met the precision criteria. Calculations based on the equation for simple random sampling of a binary population to obtain a confidence level of 95% and a relative error of 0.20 deemed a sample size of 1,276 goats sufficient to detect a rate of 7% in a population size of 2,698,636 goats (13). Population data are from the 2017 U.S. census of agriculture report (14). The rate of 7% is based on *C*. *burnetii* shedding in milk from a study in Indiana (15). The minimum sample size is further adjusted to account for clustering within herds based on sampling of 15 goats per herd according to the equation D = 1 + (*b*−1)*p*, where *b* is the number of goats sampled per herd and *p* is the intracluster correlation coefficient (13). The intracluster correlation coefficient is set at 0.06 based on data from *C*. *burnetii* in goats in Moretele (16). After accounting for *C*. *burnetii*-specific clustering an estimated 2,348 goats (1,276 × 1.84) across 157 operations (2,348/15) is deemed sufficient to obtain a confidence level of 95% and a relative error of 0.20. The subset of vaginal swabs tested (4,121 from 388 operations) exceeded this minimum.

#### Sample storage conditions

Swabs were maintained at 4°C for a median time of 7 days (range: 1–169) and shipped to the Centers for Disease Control and Prevention (CDC) on ice packs.

### Analysis of *Coxiella burnetii* DNA from vaginal swabs

#### Sample processing

Upon arrival at the CDC, swabs were placed in 800 μL of sterile PBS and vortexed for 30–60 s, followed by incubation at 35°C with shaking at 200 rpm for 1 h.

#### DNA extraction

DNA was extracted from a 200 μL aliquot and remaining volumes were stored at −80°C. DNA extractions were automated using the KingFisher Flex System in conjunction with the KingFisher Cell and Tissue DNA Kit (Thermo Fisher Scientific) according to the manufacturer’s instructions.

#### PCR analysis

Resulting eluates were analyzed for the presence of *C*. *burnetii* DNA using a quantitative TaqMan PCR assay specific for the multi-copy *IS1111* sequence as previously described (17). A total of 320 (7.5%) DNA eluates were randomly selected for testing for the presence of PCR inhibitors as previously described (17). Analysis of 234 of the 4,121 samples was deemed sufficient to obtain a confidence level of 95% with a 1% margin of error based on an expected frequency of 0.65% (unpublished observations) (Epi Info v7.2.3.1). The subset of eluates tested for inhibitors exceeded this minimum and no inhibition was evident in any of the tested samples. Samples with at least one replicate Ct value equal to or less than 40 were considered positive for *C*. *burnetii* DNA, while samples with both replicate Ct values greater than 40 were considered negative. Genotyping analysis using a rapid PCR-based method to identify single nucleotide polymorphisms (SNP) was conducted on the four samples with the highest concentration of *C*. *burnetii* DNA, based on *IS1111* Ct values; however, only one sample had sufficient *C*. *burnetii* DNA to allow for successful analysis. This analysis identifies the *C*. *burnetii* sequence type as defined by multispacer sequence typing as previously described (18).

#### Statistical analysis

Animal-level data and operation-level responses were aggregated into data sets for analysis using SAS software version 9.4 (19). Mode-based imputation was used to infer values of factors of interest where item-level non-response existed in the survey data (20, 21).

Descriptive analyses included computation of frequencies, proportions, means, and standard errors. Estimates of proportions of positive operations and goats on positive operations were computed, and univariate and multiple logistic regression models were fit using R version 4.1.1 (22), implemented within R Studio version 2022.12.0.4353 (23). Estimation was performed using the R survey package (24), which accounts for survey sampling design and weighting. These adjustments allow inference to the population of domestic goat operations with five or more goats in the states included in the study and to the goats on those operations, specifically to those does 15 months of age or older. In addition to the survey package, other R packages were used to import and manipulate data, perform estimation and modeling, and create plots (24–31).

The svyglm function from the survey package in R was used to fit weighted logistic regression models. The svyglm function fits binary response regression models to survey data, which accounts for the survey sampling design (including stratification and clustering) and survey weighting and estimates standard errors using Taylor series linearization. Sample selection weights were adjusted for nonresponse and calibrated to known population totals at Phase I, Phase II, and the vaginal swab biologics collection phase of the study at the levels of the stratification variables (region, herd size, and operation type). Univariate operation-level prevalence models were fit using a binary response variable that took on a value of 1 if the operation had a positive swab for *C*. *burnetii* and a 0 if the operation was not positive. Univariate animal-level prevalence models were fit on data from does that were on positive operations using a binary response variable that took on a value of 1 if the doe had a positive swab and 0 if the doe did not have a positive swab. Multiple logistic regression models were fit using the svyglm function as well. Risk factors that had a univariate *p* value of 0.20 or less, had sufficient cell sizes, and were biologically plausible were considered for inclusion in multiple regression model selection. Confounding relationships between potential risk factors were assessed using tests of significance of potential confounding effects on other potential risk factors, of potential confounding effects and potential risk factors on *C*. *burnetii* presence, and changes in odds ratios in multiple regression models by more than 10% (32). All possible models were ranked, and the final model was selected according to AIC (33) and were further assessed using the precision-recall F_1_ statistic (34), which estimates the ability of the model to accurately estimate the presence or absence of *C*. *burnetii* on operations in the population, as well as McFadden’s adjusted pseudo-*R*^2^ value (35), which attempts to quantify the improvement of the model over the intercept-only model. Estimated odds ratios along with their estimated 95% confidence intervals were calculated using these logistic regression models. Overall statistical significance of factors in the weighted logistic regression model were assessed using Type III Wald test *p* values (26, 36). Pairwise comparisons were made using Tukey’s Honestly Significant Difference *p* values in order to account for multiple comparisons (37) and pairwise comparison results were displayed using a capital letter coding (38). Statistical significance was assessed at the 0.05 significance level.

#### Screening for *Coxiella burnetii* growth inhibition

The select agent exempt strain of *C*. *burnetii*, Nine Mile phase II clone 4, was propagated and grown in ACCM-2 as previously described (39). Drugs were resuspended in DMSO or sterile water (gallic acid and tannic acid) at 10 mg per mL and added to the growth media at final concentrations of 10.0, 1.0, 0.1, and 0.01 μg per mL alongside a no drug control. Samples were collected on days 0 and 7, and DNA quantified by *com1* PCR as described previously (40). The quantity of *C. burnetii* was expressed as genome equivalents (GE) and the mean ± SEM were determined at days 0 and 7. Data are reported as fold change in the mean GE between days 0 and 7. The upper and lower bounds for fold change were calculated based on the mean ± SEM as described previously (39). Data are from two independent experiments.

## Results

### Sample profile

Active *C*. *burnetii* infections in the United States domestic doe goat population were analyzed by testing for vaginal shedding. A total of 4,121 vaginal swabs were tested from does across 388 operations. The proportion of samples positive for *C*. *burnetii* by PCR analysis was 1.5% (61/4121) of does and 10.3% (40/388) of operations. The mean Ct value among positives was 37.9 (SEM = 0.1919) and positive values ranged from 32.1 to 39.8. Genotyping of the most concentrated sample identified the strain as an ST8. DNA quantities of all other samples were insufficient for genotype analysis. Overall, an average of 63.9% of the does on the operation were sampled. At least half of the does were tested by PCR on 66.0% of the operations and 100.0% of the does were tested on 31.4% of the operations, while the lower and upper quartiles of the distribution of does tested on the operation were 33.3 and 100.0%, respectively. The number of does tested per operation ranged from 1 to 20 with a mean of 10.6, and lower and upper quartiles of 7 and 15 does, respectively.

### Univariate analysis of *Coxiella burnetii* animal-level prevalence among United States goat does

Univariate logistic regression modeling of factors associated with *C*. *burnetii* animal-level prevalence was conducted on goat operations positive for *C*. *burnetii*. There was no item nonresponse for the animal-level variables for animals on positive operations. This analysis included operations with at least one positive vaginal swab resulting in a total of 479 does analyzed (Table 1). Overall, an estimated 15.9% (95% CI 11.4–21.8) of does across all positive operations had a positive sample for *C*. *burnetii* while an estimated 1.8% (95% CI 1.0–3.3) of does across all operations were positive. No significant differences were observed by month of sample collection (*p* = 0.3763) or by breed (*p* = 0.3523). Pregnant does had proportionally reduced odds of shedding *C*. *burnetii* relative to both nursing does (OR = 4.5, 95% CI 1.8–11.1) and open does (not pregnant or nursing) (OR = 3.3, 95% CI 1.5–7.3). Multiple logistic regression modeling was not conducted for animal-level prevalence as the only variable in the animal-level univariate analysis to meet the threshold for inclusion (*p* < 0.2) is unlikely to accurately inform infection risk. Specifically, shedding of *C*. *burnetii* in vaginal secretions often occurs only after parturition or abortion; therefore, pregnant does may be infected but not actively shedding (2, 41).

**Table 1:** Univariate logistic regression model results for the animal-level prevalence among animals on operations positive for *Coxiella burnetii*.

Category	Animals tested (among positive operations only)		Odds ratio	*p*-value
	% (SE)^a^	% Positive^b^ (SE)^a^	PE^c^ (95% CI^d^)	
All operations		15.9 (2.6)		
Month of sample collection				
September 2019	17.2 (11.6)	17.2 (2.8)	1.4 (0.8, 2.3)	0.3763
October 2019	41.8 (14.4)	13.0 (2.0)	(referent)	
November 2019 or later	41.0 (13.5)	18.3 (5.1)	1.5 (0.7, 3.2)	
Goat breed				
Alpine	13.2 (5.3)	16.1 (5.0)	1.8 (0.6, 5.4)	0.3523
Boer	37.1 (13.0)	15.7 (2.2)	1.8 (0.7, 4.2)	
LaMancha	9.7 (3.9)	18.3 (6.6)	2.1 (0.7, 6.6)	
Nigerian dwarf	3.8 (1.9)	9.5 (3.5)	(referent)	
Nubian	2.3 (1.5)	19.3 (2.4)	2.3 (1.0, 5.4)	
Saanen	11.8 (4.5)	26.1 (17.8)	3.4 (0.5, 24.7)	
Crossbred	9.6 (3.7)	10.3 (4.5)	1.1 (0.3, 4.1)	
Other or unspecified	12.4 (5.9)	10.5 (3.9)	1.1 (0.4, 3.5)	
Doe status				
Nursing	37.4 (11.8)	21.6 (5.9)	4.5 (1.8, 11.1)	0.0019
Pregnant	24.6 (7.8)	5.8 (1.9)	(referent)	
Open	38.0 (11.5)	16.9 (2.5)	3.3 (1.5, 7.3)	

^a^Standard error. ^b^Displayed are the weighted percentage positive animals within each subcategory. ^c^Point estimate. ^d^Confidence interval.

### Univariate analysis of *Coxiella burnetii* operation-level prevalence among United States goat herds

Univariate logistic regression modeling was performed to assess management practices associated with operation-level shedding of *C*. *burnetii* across 388 operations (Table 2; Supplementary Table S1). The weighted estimate of the percentage of operations with one or more positive does was 7.8% (95% CI 4.4–13.5). No significant difference was observed by region, herd size, primary production of the operation, and primary land/facility management type between positive and negative operations. Operations reporting suspected Q fever in the herd within the previous 3 years had 119.5 times higher odds (95% CI 16.8–851.9) of testing positive for *C*. *burnetii*. Operations with wild deer, elk, or other hoof stock on the operation or adjacent property had 10.9 times higher odds (95% CI 2.8–42.9) of testing positive and operations with wild predators such as coyotes, bears, mountain lions, or wolves on the operation or adjacent property had 11.3 times higher odds (95% CI 2.5–51.7) of having a doe test positive. There was not a significant association between operations reporting other animals such as sheep, cattle, horses, chickens, dogs, cats, or raccoons on the operation or adjacent property and operation status. Operations that utilized hormones for estrus synchronization had an 8.7 (95% CI 1.3–59.0) times higher odds of being positive than operations that did not. Never or sometimes requiring handwashing after handling goats with scabs around the mouth, feet, or udder, was associated with a 26.8 (95% CI 2.2–328.4) times higher odds of being positive compared to operations that always required hand washing. Operations that had goats that temporarily left the operation and returned and isolated those goats only for a specific reason had a 24.4 (95% CI 2.7–225.4) times higher odds of being positive with *C*. *burnetii* compared to operations that routinely isolated returning goats. Operations reporting litters of kittens had 10.6 (95% CI 2.3–49.6) times lower odds of testing positive for *C*. *burnetii*. Finally, treatment for worms during the previous 12 months using tetrahydropyrimidines, high tannin concentrate plants, or diatomaceous earth had proportionally lower odds of testing positive by factors of 23.4 (95% CI 3.5–157.6), 42.5 (95% CI 7.2–250.1), and 69.1 (95% CI 11.9–401.2), respectively, compared to operations that dewormed but not with the given product.

**Table 2:** Univariate logistic regression model results for the operation-level prevalence of *Coxiella burnetii*.

Category	Operations tested		Odds ratio	*p*-value
	% (SE)^a^	% Positive^b^ (SE)^a^	PE^c^ (95% CI^d^)	
All operations		7.8 (2.2)		
Region				
West	32.7 (2.0)	8.8 (3.6)	1.2 (0.4, 4.1)	0.7442
East	67.3 (2.0)	7.3 (2.8)	(referent)	
Herd size (number of goats and kids)				
Small (5–19)	58.6 (3.5)	9.4 (3.5)	2.3 (0.7, 7.7)	0.3367
Medium (20–99)	36.1 (3.4)	4.4 (2.0)	(referent)	
Large (100 or more)	5.4 (1.1)	12.3 (8.5)	3.0 (0.5, 18.7)	
Primary production of operation				
Meat	55.1 (3.7)	7.3 (3.2)	1.1 (0.3, 4.1)	0.8486
Dairy	17.6 (2.4)	6.7 (2.9)	(referent)	
Other	27.3 (3.6)	9.5 (4.5)	1.5 (0.4, 5.6)	
Primary land/facility type				
Open/Fenced range	38.0 (3.7)	12.6 (4.9)	3.0 (0.9, 10.6)	0.2042
Fenced farm	49.3 (4.0)	4.5 (2.0)	(referent)	
Outdoor dry lot/Indoors	12.7 (2.4)	6.2 (3.6)	1.4 (0.3, 5.9)	
Any bred does aborted in the previous 12 months				
Yes	19.5 (3.1)	6.6 (3.3)	(referent)	0.7231
No or no bred does	80.5 (3.1)	8.1 (2.6)	1.3 (0.4, 4.3)	
Q Fever suspected in herd in previous 3 years^k^				
Yes	1.0 (0.6)	90.0 (8.6)	119.5 (16.8, 851.9)	<0.0001
No	99.0 (0.6)	7.0 (2.2)	(referent)	
Domestic goats on an adjacent property with fence-line^k^				
Yes	8.0 (2.0)	1.6 (1.4)	(referent)	0.0681
No	92.0 (2.0)	8.3 (2.4)	5.6 (0.9, 36.1)	
Wild deer, elk, or other hoof stock on this operation or on adjacent property^i^				
Yes	80.0 (3.1)	9.5 (2.8)	10.9 (2.8, 42.9)	0.0007
No	20.0 (3.1)	1.0 (0.6)	(referent)	
Wild predators (e.g., coyotes, bears, mountain lions, and wolves) on this operation or on adjacent property^i^				
Yes	82.5 (3.2)	9.3 (2.7)	11.3 (2.5, 51.7)	0.0020
No	17.5 (3.2)	0.9 (0.6)	(referent)	
Outdoor or indoor/outdoor domestic cats on this operation^l^				
Yes	74.0 (3.7)	9.3 (2.8)	2.8 (0.7, 12.1)	0.1669
No	26.0 (3.7)	3.5 (2.3)	(referent)	
Feral or stray cats on this operation				
Yes	53.8 (3.7)	5.9 (2.5)	(referent)	0.3544
No	46.2 (3.7)	10.0 (3.7)	1.8 (0.5, 5.8)	
Litters of kittens on this operation^l^				
Yes	20.2 (3.1)	1.0 (0.7)	(referent)	0.0028
No	79.8 (3.1)	9.5 (2.8)	10.6 (2.3, 49.6)	
Were hormones used for estrus synchronization^k^				
Yes	1.7 (0.8)	40.5 (22.1)	8.7 (1.3, 59.0)	0.0263
No or no bred does	98.3 (0.8)	7.2 (2.2)	(referent)	
Were any goats or kids permanently added to the operation in the previous 12 months^i^				
Yes	35.0 (3.7)	11.7 (4.6)	2.2 (0.7, 7.0)	0.1878
No	65.0 (3.7)	5.7 (2.2)	(referent)	
Did any goats or kids temporarily leave the operation and return in the previous 12 months^j^				
Yes	25.5 (3.2)	14.5 (6)	2.9 (0.9, 9.9)	0.0881
No	74.5 (3.2)	5.5 (2.1)	(referent)	
Any goats dewormed in the previous 3 years^j^				
Yes	94.9 (1.7)	8.0 (2.3)	1.8 (0.3, 12.4)	0.5567
No	5.1 (1.7)	4.6 (4.1)	(referent)	
During the previous 12 months, when goats or kids temporarily left and returned, were they isolated for any period of time prior to re-introduction to the herd^i^				
Never	14.5 (2.9)	10.7 (6)	7.4 (0.9, 59.9)	0.0330
Only for specific reason	7.3 (2.4)	28.3 (15.2)	24.4 (2.7, 225.4)	
Routinely	3.6 (1.1)	1.6 (1.3)	(referent)	
N/A^e^	74.5 (3.2)	5.5 (2.1)	3.6 (0.6, 22.9)	
Years of experience owning or managing goats^i^				
0–5	19.9 (3.4)	2.6 (2)	(referent)	0.0521
6–10	33.7 (3.7)	3.3 (1.7)	1.3 (0.2, 9.0)	
11–20	24.4 (3.4)	13.4 (6.2)	5.9 (0.9, 40.2)	
21 or more	21.9 (3.3)	13.3 (5.7)	5.9 (0.9, 37.2)	
Months in which majority of kids were born in the previous 12 months^i^				
Spring (Mar, Apr, May)	45.3 (4.0)	10.9 (3.8)	3.7 (0.9, 15.2)	0.1409
Fall (Oct, Nov, Dec)	10.5 (2.4)	13.6 (8.6)	4.7 (0.7, 31.0)	
N/A^f^	44.2 (4.1)	3.2 (1.9)	(referent)	
During the previous 12 months, how often were hands washed with soap and water after touching goats with scabs^i^				
Always	7.4 (2.1)	1.2 (0.9)	(referent)	0.0179
Sometimes or never	2.3 (1.1)	24.6 (19.3)	45.5 (3.1, 675.6)	
N/A^g^	90.3 (2.4)	7.9 (2.4)	7.0 (1.4, 34.6)	
During the previous 12 months, treated for worms using high tannin concentrate plants^k^				
Yes	6.1 (1.6)	0.2 (0.2)	(referent)	0.0001
No	82.3 (3.0)	9.1 (2.6)	42.5 (7.2, 250.1)	
N/A^h^	11.6 (2.6)	2.2 (1.8)	9.6 (0.9, 101.1)	
During the previous 12 months, treated for worms using diatomaceous earth^k^				
Yes	9.5 (2.2)	0.2 (0.1)	(referent)	<0.0001
No	78.9 (3.2)	9.5 (2.7)	69.1 (11.9, 401.2)	
N/A^h^	11.6 (2.6)	2.2 (1.8)	14.9 (1.4, 154.3)	
During the previous 12 months, treated for worms using tetrahydropyrimidines^k^				
Yes	3.5 (1.4)	0.4 (0.4)	(referent)	0.0026
No	84.9 (3.0)	8.9 (2.6)	23.4 (3.5, 157.6)	
N/A^h^	11.6 (2.6)	2.2 (1.8)	5.5 (0.5, 62.8)	
During the previous 12 months, treated for worms using avermectins^k^				
Yes	61.5 (3.8)	11.9 (3.5)	17.3 (4.6, 64.6)	0.0001
No	26.9 (3.4)	0.8 (0.5)	(referent)	
N/A^h^	11.6 (2.6)	2.2 (1.8)	2.9 (0.4, 21.5)	
During the previous 12 months, provided additional protein supplement to increase resistance as a part of an internal parasite control program^i^				
Yes	35.9 (3.7)	4.3 (2.3)	(referent)	0.1854
No	64.1 (3.7)	9.7 (3.2)	2.4 (0.7, 8.6)	

^a^Standard error. ^b^Displayed are the weighted percentage positive operations within each subcategory. ^c^Point estimate. ^d^Confidence interval. ^e^No goats temporarily left the operation and returned. ^f^Other months or no kids born on the operation. ^g^No goats had signs of sore mouth in the previous 12 months. ^h^No goats dewormed in the previous 3 years or the previous 12 months. ^i^Included in multiple regression modeling. ^j^Included in multiple regression modeling but duplicate of another variable. ^k^Omitted from multiple regression modeling due to small sample sizes. ^l^Omitted from multiple regression modeling due to epidemiological reason.

### Effect of select tetrahydropyrimidines and tannins on growth of *Coxiella burnetii in vitro*

**Figure 1:**
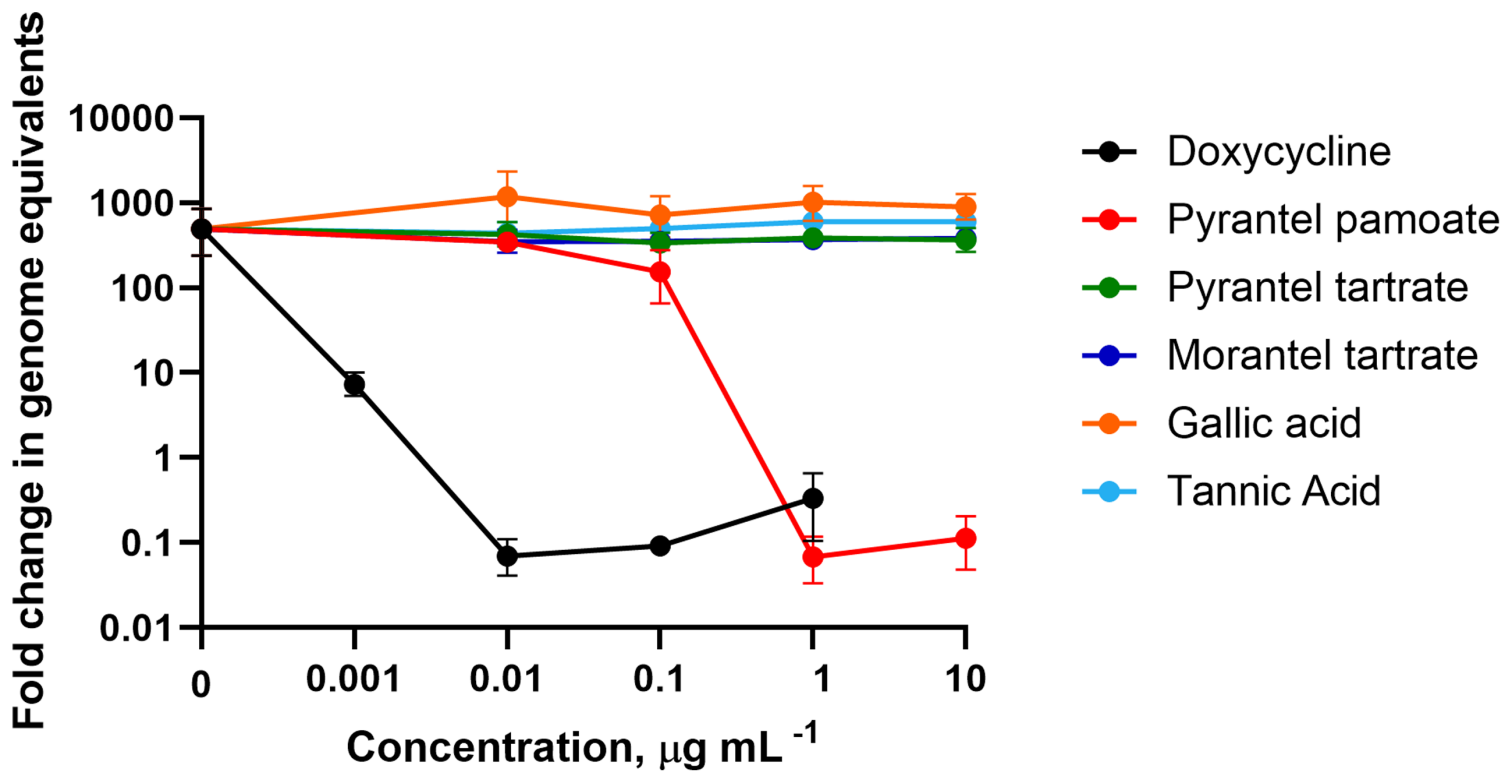
Susceptibility of *Coxiella burnetii* to hydrolysable tannins and tetrahydropyrimidines. *Coxiella burnetii* was grown for 7 days in ACCM-2. Data demonstrate the fold change of the mean genome equivalents for day 7 relative to day 0. Error bars represent the upper and lower bounds of fold change, which were calculated based on the SEM of the genome equivalents. Data are from two independent experiments.

As operations that treated for worms during the previous 12 months using tetrahydropyrimidines or high tannin concentrate plants were associated with decreased shedding of *C*. *burnetii*, we sought to examine the effect of these compounds on the growth of *C*. *burnetii in vitro*. To this end, we cultured *C*. *burnetii* in the presence of tetrahydropyrimidines (pyrantel pamoate, pyrantel tartrate, and morantel tartrate) as well as the hydrolysable tannins, tannic acid, and gallic acid, at concentrations up to 10 μg per mL. Pyrantel pamoate demonstrated an inhibitory effect on the growth of *C*. *burnetii* as low as 1 μg per mL (1.68 μM) (Figure 1). For comparison, the MIC for doxycycline, the recommended treatment option for humans, is 0.01 μg per mL (22.5 nM).

### Multiple regression analysis of *Coxiella burnetii* operation-level prevalence among United States goat herds

The effects of imputation on the final results were minimal. There were 36 observations that had mode-imputed values across 11 of the 48 operation-level variables considered in Supplementary Table S1. The range in the number of missing values for those 11 variables was 1–12. None of the percentages of operations changed by more than 5.1% (2.2 percentage points), the greatest change being in the majority of kidding that occurred in the spring (Mar, Apr, May), which changed from 45.3% (SE = 4.0) to 43.1% (SE = 4.0). None of the percent of positive operations changed by more than 7.8% (0.9 percentage points), the largest being in the majority of kidding in spring (Mar, Apr, May), which changed from 10.9% (SE = 3.8) to 11.8% (SE = 4.2). Only one of the *p* values changed significance at the 0.05 level, for years of experience raising goats, which changed from 0.0521 to 0.0465.

A model summary for the multiple logistic regression model fit to model the operation-level prevalence of *C*. *burnetii* is included in Table 3. The only risk factor that was statistically significant at the 0.05 significance level after accounting for the other factors included in the model was the presence of wild predators (e.g., coyotes, bears, mountain lions, and wolves) on the operation or adjacent property (OR = 9.0, 95% CI 1.3–61.6, *p* value = 0.0248). Five other variables were statistically significant at the 0.10 significance level but not the 0.05 significance level. These included the presence of wild deer, elk, or other hoof stock on the operation or adjacent property (OR = 8.1, 95% CI 0.9–72.8, *p* value = 0.0608), frequency of hand washing after touching goats with scabs (OR = 1.9, 95% CI 0.1–33.9 and OR = 6.0, 95% CI 0.5–36.4 for operations that sometimes or never did so and for operations with no goats with signs of sore mouth, respectively, compared to operations that always washed hands, *p* value = 0.0884), whether goats and kids that temporarily left the operation and returned were isolated after returning to the operation (OR = 3.7, 95% CI 0.7–20.4 and OR = 10.3, 95% CI 1.2–85.6 for operations that never isolated and for operations that isolated only for a specific reason, respectively, compared to operations that isolated routinely or did not have any goats temporarily leave and return, *p* value = 0.0891), the length of time that goats had been managed (OR = 1.6, 95% CI 0.1–23.3, OR = 6.5, 95% CI 0.5–80.2, and OR = 11.3, 95% CI 0.7–196.0, for operations that owned or managed goats for 6–10, 11–20, and 21 or more years, respectively, compared to operations that did so for 0–5 years, *p* value = 0.0990), and the months in which the majority of kids were born (OR = 3.5, 95% CI 0.8–14.5 and OR = 6.5, 95% CI 0.5–80.2 for operations that primarily kidded in spring and fall, respectively, compared to operations that kidded in other months or had no kids born on the operation, *p* value = 0.0550). The McFadden’s adjusted pseudo-*R*^2^ value for this model was 0.397, approximately, and the *F*_1_ score was 0.286.

**Table 3:** Multiple logistic regression model results for the operation-level prevalence of *Coxiella burnetii*.

Parameter	*p*-value^d^	Parameter level	Significant differences	Odds ratio	95% CI
Intercept					
Wild deer, elk, or other hoof stock on this operation or on adjacent property	*0*.*0608*	Yes		8.1	(0.9, 72.8)
		No		(referent)	
Wild predators (e.g., coyotes, bears, mountain lions, and wolves) on this operation or on adjacent property	**0.0248**	Yes	A	9.0	(1.3, 61.6)
		No	B	(referent)	
During the previous 12 months, how often were hands washed with soap and water after touching goats with scabs	*0*.*0884*	Always		(referent)	
		Sometimes or Never		1.9	(0.1, 33.9)
		N/A^a^		6.0	(0.5, 36.4)
Were any goats or kids permanently added to the operation in the previous 12 months	0.1314	Yes		2.5	(0.8, 8.5)
		No		(referent)	
During the previous 12 months, when goats or kids temporarily left and returned, were they isolated for any period of time prior to re-introduction to the herd	*0*.*0891*	Never		3.7	(0.7, 20.4)
		Only for specific reason		10.3	(1.2, 85.6)
		N/A^b^		(referent)	
Years of experience owning or managing goats	*0*.*0990*	0–5		(referent)	
		6–10		1.6	(0.1, 23.3)
		11–20		6.5	(0.5, 80.2)
		21 or more		11.3	(0.7, 196.0)
Months in which the majority of kids were born in the previous 12 months	*0*.*0550*	Spring (Mar, Apr, May)		3.5	(0.8, 14.5)
		Fall (Oct, Nov, Dec)		6.5	(1.2, 36.0)
		N/A^c^		(referent)	
Region	0.5909	West		1.5	(0.4, 6.0)
		East		(referent)	
Herd size (number of goats and kids)	0.1730	Small (5–19)		3.7	(0.9, 14.6)
		Medium (20–99)		(referent)	
		Large (100 or more)		1.7	(0.5, 6.4)
Primary production of the operation	0.2673	Meat		1.6	(0.2, 10.3)
		Dairy		(referent)	
		Other		3.7	(0.6, 23.2)

^a^No goats had signs of sore mouth in the previous 12 months. ^b^Routinely or No goats temporarily left the operation and returned. ^c^Other months or no kids born on the operation. ^d^Bolded values *p* < 0.05, Italicized values *p* < 0.10.

## Discussion

To our knowledge, this is the first large scale report of *C*. *burnetii* infection across the United States goat population. Active shedding of *C*. *burnetii* was investigated for 4,121 doe goats, which demonstrated 1.5% positivity at the time of sampling. The quantity of *C*. *burnetii* being shed was low, which is likely influenced by the timing of sample collection. The bulk of samples were collected in October and November and positivity and bacterial loads would likely have been higher in the spring, when most operations have kid goats born. In this study, animal-level univariate analysis found pregnant does had reduced odds of testing positive for *C*. *burnetii*. Shedding in vaginal secretions often only occurs after parturition or abortion and the bacterial load is typically highest immediately post-kidding (2, 41). The lower percentage of pregnant does shedding the bacteria is likely highlighting the shedding dynamics of *C*. *burnetii* rather than a lower infection rate in pregnant does but underscores the fact that targeted sampling following the kidding seasons may increase positivity. Given the fact that testing occurred outside of kidding season and did not target high risk goats, the data suggest widespread infection of goat herds across the United States at any given time underscoring the importance of the disease and the potential risk to goat producers and the public.

*Coxiella burnetii* seroprevalence has been reported in goats in the United States with herd-level prevalence ranging from 11.5, 21.0, and 41.6% (15, 42, 43); however, given that seropositivity can persist for years after infection and infected animals may be seronegative, evaluation of active infections by molecular analysis of *C*. *burnetii* shedding is important (44). Studies investigating *C*. *burnetii* shedding prevalence are limited, particularly for goats in the United States. Small, localized studies found shedding prevalence ranging from 0% in fecal samples from agricultural fairs in Iowa to 7% in milk from Indiana (15, 45). Herein, we identified 61 does positive for shedding *C*. *burnetii* across 10.3% of the tested operations (7.8% weighted, SE = 2.2), which is in line with the observations in Indiana, but is lower than that observed in goats worldwide. Shedding was observed in 60% of sheep and goat herds in Algeria, 51.16% of goat herds in Poland, and between 16.6 and 56.4% in Iran (46–49). Shedding in dairy goat herds in Belgium decreased from 12% in 2009 to 6.3% in 2012 and 6% in 2019 only after the introduction of mandatory vaccination in 2011 (50, 51). Curiously, the shedding prevalence of goat herds in the United States observed in the current study is better aligned with that of vaccinated herds despite the inability to vaccinate goats against *C*. *burnetii* in the United States. These differences are likely multifactorial but may involve the presence of different *C*. *burnetii* genotypes. The ST61 and ST18 genotypes have been reported in goat herds in Poland, ST61 and ST62 genotypes have been reported in Iran, ST61 and ST33 genotypes have been reported in goats in Belgium, while the ST8 genotype is consistently associated with goats in the United States (47, 52–55). Only one sample was concentrated enough in the present study to conduct genotyping analysis and indeed it belonged to the ST8 genogroup. Murine *in vivo* studies have found strains from the ST8 genogroup to be less virulent than other genotypes which may account for the reduced shedding prevalence observed in United States goat herds (52, 56–58). Despite this, ST8 strains can cause abortions in goats and chronic Q fever in humans (52, 53).

Operations reporting the presence of wild elk, deer, and other hoof stock were more likely to test positive for *C*. *burnetii* by univariate analysis, while the presence of wild predators on the operation or adjacent property was significantly associated with greater odds of testing positive by both univariate and multiple logistic regression modeling. These findings highlight the possible role for wild animals as *C*. *burnetii* reservoirs. Antibodies against *C*. *burnetii* have been detected in coyotes at 78% and foxes at 55% and viable *C*. *burnetii* has been isolated from tissues of both species (59). *Coxiella burnetii* seroprevalence of wild deer in parts of the United States has been documented as high as 22% (59, 60). *Coxiella burnetii* infection in red deer can lead to abortions, shedding, and transmission to humans (61). The proximity of wild predators and deer to domestic animals and humans highlights the need to further examine their role in the maintenance and transmission of *C*. *burnetii*.

Another factor associated with greater odds of *C*. *burnetii* infection in this study was operations utilizing hormones for estrus synchronization. In experimental settings, sex hormones have been shown to influence the host response to *C*. *burnetii* infection (62, 63). Progesterone, which is commonly administered for estrus synchronization in goats has been shown to inhibit replication of *C*. *burnetii* (64). The findings herein, demonstrate that administration of hormones for estrus synchronization does not have a protective effect in this setting, rather *C*. *burnetii* may be more likely to spread due to large quantities of *C*. *burnetii* being introduced into the environment at once from a more concentrated kidding season. Furthermore, shared equipment during the estrus synchronization process could promote the spread of *C*. *burnetii* throughout the herd. The influence of other management practices among operations that utilize estrus synchronization cannot be ruled out.

Herds treated with high tannin concentration plants, diatomaceous earth, or tetrahydropyrimidines were less likely to test positive for *C*. *burnetii*. The antimicrobial properties of tannins are well documented and the tannin containing forage, sainfoin, has been shown to decrease fecal shedding of *Escherichia coli* in cattle (65–69). Although the hydrolysable tannins, tannic acid and gallic acid, had no direct effect on growth of *C*. *burnetii in vitro* (Figure 1), the activity of condensed tannins remains to be determined. Diatomaceous earth possesses electromagnetic properties that bind bacteria (70). Although inhalation is the major route of infection by *C*. *burnetii*, infection via the oral route is possible (56, 58). Therefore, treatment with diatomaceous earth may serve to reduce *C*. *burnetii* in the gastrointestinal tract and possibly a reduction in the aerosol burden as *C*. *burnetii* complexed with the diatomaceous earth are excreted into the environment. Pyrantel pamoate, a tetrahydropyrimidine anthelmintic, can inhibit growth of *C*. *burnetii in vitro* as demonstrated in Figure 1 (71). This effect has only been characterized for the lab attenuated Nine Mile Phase II strain (ST16) of *C*. *burnetii*. As such, the efficacy of pyrantel pamoate on virulent or goat-associated strains of *C*. *burnetii* remains to be determined. The usefulness of these potential treatments for *C*. *burnetii* infection control in ruminants should be further explored; however, in the absence of further research, off-label use of anthelmintics should be restricted to avoid exacerbating resistance.

This study highlighted two biosecurity measures that goat producers may consider employing to reduce *C*. *burnetii* shedding on the operation. Operations requiring handwashing after handling goats with scabs, were less likely to test positive for *C*. *burnetii*. While *C*. *burnetii* does not cause scabs, frequent handwashing likely reduces the spread of a variety of pathogens. Secondly, routine isolation of goats returning to the operation was associated with reduced shedding. Therefore, goat producers should consider incorporating these strategies, along with other biosecurity measures into their management practices.

Operations reporting litters of kittens were associated with decreased shedding of *C*. *burnetii*, which is surprising given that exposure to parturient cats has been associated with outbreaks of Q fever (72–75). Furthermore, *C*. *burnetii* positivity in farm cats has been shown to be associated with infected ruminant farms (76). Interestingly, the presence of cats (domestic or feral) on the operation was not significantly associated with *C*. *burnetii* shedding in goats (Table 2). *Coxiella burnetii* has been detected in feline reproductive tissues and is suspected of causing feline reproductive health issues (75, 77, 78). Perhaps cats are less likely to give birth to healthy kittens on operations where *C*. *burnetii* is present; however, additional research is needed to draw any conclusions regarding the effect of *C*. *burnetii* on feline reproductive health. It is not clear if parturition in other animals such as dogs would be associated with *C*. *burnetii* shedding in goats as this information was not captured in this study.

No association was found with regard to location, herd size, and primary production of the operation, which highlights the widespread vulnerability of the goat industry to this disease. Given the rise in popularity of goats in the United States and the number of operations experiencing infection, the development and implementation of prevention and treatment strategies for *C*. *burnetii* infected goat farms is important to protect goat producers and public health. In the United States, there are currently no approved Q fever vaccines or therapies to prevent or eradicate *C*. *burnetii* from goat herds or their environment. However, this study highlights potential practices that can be implemented to identify high risk goats and mitigate exposure and spread, such as limiting visitor contact with kidding does and preweaned kids, ensuring handwashing protocols for visitors, routine isolation of goats returning to the operation, and minimizing contact with wildlife when feasible. However, just as important, this study highlighted that producers are aware of *C*. *burnetii* in their herds as shedding was associated with operations that suspected Q fever; therefore, this could be capitalized on should effective therapies be identified. However, the percentage of operations that suspected *C*. *burnetii* in their herds in the previous 3 years (1.0%, SE = 0.6) was much lower than the estimated percentage of operations that tested positive at the time of the study (7.8%, SE = 2.2) suggesting that raising awareness of this important disease among goat producers is needed.

In conclusion, *C*. *burnetii* infected goat herds are more common across the United States than reported outbreaks would suggest, highlighting the need for effective prevention and treatment strategies for these operations and continued surveillance to reduce public health risk. This study highlights that some producers are aware of *C*. *burnetii* infection in the herd, however there is still room for improvement. Therefore, future public health prevention measures should focus on the development of mitigation strategies alongside awareness campaigns. This study also highlights *C*. *burnetii* potential therapies that should be further investigated as they are already approved for use in goats. However, caution should be taken, as using anthelmintics off-label or inappropriately may promote anthelmintic resistance. Any treatment should be performed under the advisement of a veterinarian with valid veterinary-client-patient-relationship (VCPR). Finally, the analysis demonstrates a need to investigate the role of potential wildlife reservoirs, particularly wild predators, in the maintenance and transmission of *C*. *burnetii*.

## Data availability statement

The data that support the findings of this study are available upon reasonable request from the corresponding author; however, restrictions apply as some data are not available due to the sensitive nature of the research. Data availability will be at the discretion of the USDA Animal and Plant Health Inspection Service (APHIS). Requests to access the datasets should be directed to HM, Halie.Miller@cdc.hhs.gov.

## Ethics statement

Ethical approval was not required for the studies involving animals in accordance with the local legislation and institutional requirements because as a federal facility conducting research on owned livestock, the USDA Animal and Plant Health Inspection Service (APHIS) is not required to register under the U.S. Animal Welfare Act. However, as the agency that administers the Animal Welfare Act, USDA APHIS staff ensure that all animal treatment that is part of the National Animal Health Monitoring System is in compliance with the Animal Welfare Act. Written informed consent was obtained from the owners for the participation of their animals in this study.

## Author contributions

HM: Conceptualization, Formal analysis, Investigation, Methodology, Writing – original draft, Writing – review & editing. MB: Formal analysis, Methodology, Writing – review & editing. RP: Investigation, Writing – review & editing. RÁ-A: Investigation, Writing – review & editing. CC: Conceptualization, Writing – review & editing. CS: Investigation, Writing – review & editing. NU: Resources, Writing – review & editing. AW: Resources, Writing – review & editing. CB: Formal analysis, Methodology, Writing – review & editing. KM: Conceptualization, Resources, Supervision, Writing – review & editing. GK: Conceptualization, Methodology, Project administration, Resources, Supervision, Writing – review & editing.

## Supplementary material

SUPPLEMENTARY TABLE S1 Univariate logistic regression model results for all variables considered for analysis.
